# Strengthening the Somaliland health system by integrating public and private sector family medicine

**DOI:** 10.4102/phcfm.v13i1.3049

**Published:** 2021-09-16

**Authors:** Muna M. Mahfud, Fathia M. Nour, Hodan J. Abdi, Sabah M. Muse, Tim Fader

**Affiliations:** 1 Department of Family Medicine, Amoud University, Borama, Somalia

**Keywords:** public–private, family medicine, family medicine training, Somaliland, universal health coverage, district hospital, essential package of health services, primary health care

## Abstract

Four family physicians, who received their specialty training at Amoud University in Somaliland, organised a practice together that uses informal public–private partnerships to optimise their clinical care and teaching. Their experience offers insights into public–private partnerships that could strengthen the country’s healthcare system.

## Background

Civil war broke out in Somalia in 1988. By 1991, the clans in the northwest of the country declared independence and formed a new country, Somaliland. Whilst the rest of the world does not recognise it as an independent country, Somaliland has developed relative peace and stability in contrast to the rest of Somalia. The estimated population of Somaliland is 3.8 million, with 45% population living in rural areas. Farming livestock is the backbone of the economy. Life expectancy is 55 years and the maternal mortality ratio is 396 per 100 000 live births.^1^

The Somaliland health system is based on an Essential Package of Health Services (EPHS), which the government adopted in 2009. The EPHS describes four levels of service delivery: the primary health unit, health centre, primary hospital (also known as the first referral hospital or district hospital) and regional hospital. The primary hospital is meant to be served by generalist doctors whilst the regional hospital is organised according to traditional hospital specialties.^2^

Amoud University, Somaliland’s first institution of higher education, was founded in 1998 in Borama, a town with a population of about 150 000. Amoud Medical School started in 2000. In 2012, four United States (US) trained doctors partnered with Amoud University to establish a family medicine training programme as a master’s degree (MFamMed). At the time there were no specialty training programmes for doctors in the country. During their three years of training family medicine registrars become competent to accomplish two main tasks: firstly, to provide comprehensive, holistic care at the level of the primary hospital, and secondly, to work alongside teams in the hospital’s catchment area to provide primary health care. Since the start of the programme 29 family medicine specialists have graduated.

In the Borama District public sector there are 13 health centres, one functional primary hospital and one 377-bed regional hospital. Because there is only one primary hospital in the system, the staff of most health centres refer patients directly to the regional hospital when the patients need higher level care.

In 2020, the Somaliland government implemented a policy to provide free care for obstetrics patients delivering in government health centres or at the regional hospital, if the patient is referred there from a health centre. There is no mechanism to include the private sector in this service.

The first four authors of this article are women who became friends whilst studying in medical school at Amoud. After graduation from medical school they joined the family medicine training programme and finished it in 2015 and 2016. By 2017, they had established a practice that informally brought together the public university, the public regional hospital, their own private hospital and the public primary care system.

Amoud University initially employed them as clinical instructors of medical students and interns at the public regional hospital. Later the university increased their teaching responsibilities by appointing them as either full or adjunct faculty members of the family medicine training programme, which uses the regional hospital as its main teaching hospital. Muna M. Mahfud is the director of family medicine training.

When they began work for the university at the regional hospital, the hospital had only two specialists. The hospital soon appointed them to clinical governance positions: F.M.N. is the hospital medical director and S.M.M. is in charge of the adult medicine service. H.J.A. founded the hospital’s neonatal intensive care unit (NICU) in 2017. This is the only NICU in the region. In 2020, the NICU had 264 admissions and an 85% survival rate. H.J.A. remains in charge of the service.

The fifth author of this report (T.F.) taught family medicine from 2012 to 2015. He then left Somaliland and returned in 2021. The improvements in healthcare at the regional hospital are remarkable. The family medicine faculty members and registrars brought to the hospital evidence-based standards of care, professionalism, morning report, morbidity and mortality review, improved documentation, daily rounds, regular teaching and dependable backup at night and on weekends.

Because of the specialist compartmentalisation of the regional hospital, the four family physicians were not able to practice comprehensive family medicine. In 2017, there were no primary hospitals in the Borama public sector at all. Thus, the four women decided to form a legal partnership and open their own private hospital.

They rented adjacent houses in an underserved area of town and set up inpatient and outpatient services including laboratory, ultrasound and pharmacy. They advertised their hospital through local television for two months and through patient education articles on a Facebook page for a year. They established a graduated fee-for-service billing system that facilitated access to their care by the poor in the community. They connected with nearby health centres and offered to care for their complicated patients so that they would not need to be referred to the regional hospital.

They opened all services on 7 October 2017. On the first day, they had to do a C-section on a midwife from a village in Ethiopia. Mother and baby did fine, and the relatives were surprised that women could do surgery. Box 1 shows characteristics of Awdal Hospital for the year 2020.

BOX 1 Awdal hospital 2020.

**Table:** 

Beds	40
Admissions	637
Outpatient visits	1927
Outpatient visits for non-communicable disease	35% – 40%
Deliveries	204
Caesarian sections	48

## Discussion

The public sector health system in Borama district does not function as envisioned by the EPHS. Because of a lack of public sector primary hospitals, staff at health centres refer patients directly to the regional hospital. However, there are now 10 private primary hospitals in Borama district. The government could improve the delivery of health services in Borama district by contracting private primary hospitals to fill the gap between health centres and the regional hospital. Contracting private sector services is a main goal of the Somaliland Ministry of Health Development.^3^

The government policy to provide obstetric care free of charge is a reasonable strategy to decrease maternal and neonatal mortality by increasing facility-based deliveries. The government could broaden the impact of this policy by including the option to deliver at approved private primary hospitals. This change would increase accessibility of care and would lead to more appropriate use of the regional hospital.

Because of a lack of specialists in the country, the regional hospital fills its staffing gaps with family medicine doctors. This approach has had good results at the regional hospital and is a reasonable short-term answer to the shortage of specialists. Ultimately, however, as other specialists become available for the regional hospital, the health system will function better with family physicians working at the primary hospital. This is the goal of the family medicine training programme and is consistent with the most common deployment of family physicians in sub-Saharan Africa.^4^

Whilst the same four doctors apply the same standards of care and spend about the same amount of time working in the public and private hospitals, they know that their impact on patient care is greater in their own hospital. Why is this? The answer is multifactorial, but this group believes the main reason is that they have administrative authority over the structure and process of care in their private hospital, but not in the public hospital. Whilst they have positions of responsibility in the public hospital, they lack the authority to carry out those responsibilities. Having appropriate authority to accomplish their clinical governance responsibilities would increase their impact on healthcare at the public hospital.

## Conclusion

The experience of these four family physicians demonstrates the benefits of informal public–private partnerships in clinical practice, as well as opportunities for improved patient care and broader access to care by further developing such partnerships.
